# The Use of Low-Rejection Nanofiltration Membranes as a Tool to Simplify Pretreatment, Escape Scaling and Radically Increase Recoveries

**DOI:** 10.3390/membranes15040096

**Published:** 2025-03-25

**Authors:** Alexei G. Pervov, Dmitry Spitsov, Anna Kulagina, Htet Zaw Aung

**Affiliations:** Department of Water Supply, Moscow State University of Civil Engineering, 26, Yaroslaskoye Highway, 129337 Moscow, Russia; spitsovdv@mgsu.ru (D.S.); anya.k.11@yandex.ru (A.K.); newmoon68109@gmail.com (H.Z.A.)

**Keywords:** reverse osmosis, nanofiltration, surface (storm) wastewater, drainage wastewater, membrane rejection, reduction in concentrate discharge, scaling on membranes, membrane fouling, environmental damage

## Abstract

This article describes the results of research to develop a new technology to treat storm and drainage water generated on a territory of industrial enterprises and reuse it as a feed water for boiler feed and steam generation. To develop such a system, it is necessary to resolve issues related to pretreatment, scaling, and fouling, as well as to provide a minimal discharge in the company’s sanitation network. Principles of the new approach to reach high calcium removal are based on the use of two or three stages of low-pressure nanofiltration membranes instead of the conventional facilities that contain one stage of reverse osmosis membranes. High permeability, low pressure, high recovery, and reduced reagent consumption provide an economic effect. The technology uses low-rejection membranes “nano NF” developed and produced by “Membranium Co.” (Vladimir, Russia). In the article, the results of investigations on the evaluation of scaling rates in membrane modules and rates of homogeneous crystallization in concentrate flow are presented. Processing these results enables us to detect recovery values when scaling begins on the membrane surface as well as to determine the maximum recovery value for the beginning of homogenous nucleation in the concentrate flow.

## 1. Introduction

**Figure 1:**
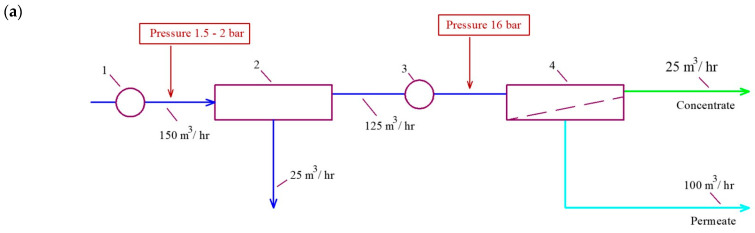

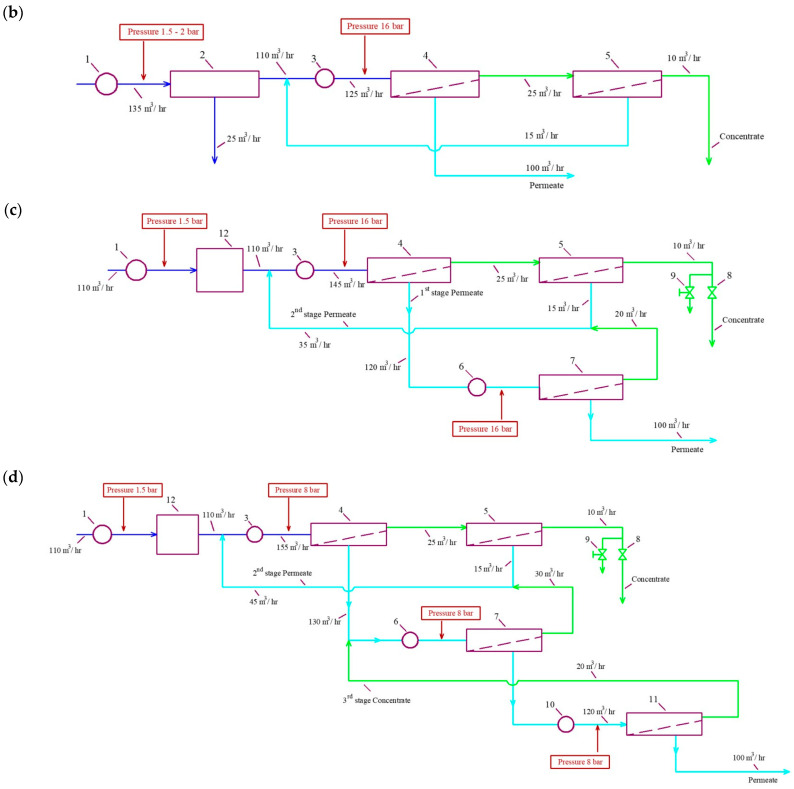
Flow diagrams of reverse osmosis and nanofiltration membrane facilities: (**a**) conventional scheme with pretreatment and concentrate discharge; (**b**) the scheme with reduced concentrate discharge due to the use of a nanofiltration membrane unit on the concentrate stream; (**c**) the new double-stage technological scheme with simplified pretreatment and concentrate reduction using the “open channel” membrane modules; (**d**) the new three-stage membrane scheme using nanofiltration membranes to produce low TDS and softened water; 1—booster pump for the feed water; 2—pretreatment system; 3—high-pressure pump for the first membrane stage; 4—first stage membrane modules; 5—nanofiltration membrane modules to reduce first stage concentrate flow; 6—second stage high-pressure pump; 7—second stage membrane modules; 8—pressure regulation valve; 9—hydraulic flushing valve; 10—third stage high-pressure pump; 11—third stage membrane modules.

Currently, the application field of membrane technologies in ecological problem-solving is growing. To improve the efficiency of water treatment systems, research is underway in the field of scaling and fouling control [1]. This article is devoted to finding a solution to the problem of wastewater reuse for industrial enterprises [2,3,4,5] and developing an optimal technological solution to produce boiler feed quality water from rainwater (surface wastewater) and drainage water [6,7,8,9,10]. The existing water treatment systems for boiler feed are based on flocculation and filtration for pretreatment and further softening through the use of the sodium–cationite ion exchange process [11,12,13]. At present, reverse osmosis technology is widely applied for boiler feed production within power industries, seawater desalination, industrial use, etc. Meanwhile, serious problems still exist that are connected with pretreatment equipment, high color removal, the use of antiscalants, the selection of cleaning chemicals, as well as concentrate discharges’ handling and utilization [14,15,16,17]. For the case presented in this article, the authors have developed a new approach to solve the abovementioned problems, such as the elimination of pretreatment and antiscalant purchase as well as the reduction in concentrate discharges [5,17]. The conventional approach to developing a reverse osmosis water treatment facility is demonstrated in Figure 1a. Above the pretreatment equipment investments, a substantial payment is required to cover reagent costs to purchase antiscalants and cleaning chemicals to remove foulants accumulated on the membrane surface. To reduce concentrate discharge, a new tool using low-rejection nanofiltration membranes is applied. The use of nanofiltration membranes allows us to solve two problems, such as increasing recovery and avoiding intensive scaling. Membrane modules tailored with low-rejection nanofiltration membranes demonstrate lower scaling propensities than reverse osmosis modules [2]. Figure 1b shows a water balance flow diagram of a reverse osmosis facility with reduced concentrate discharge that contains a second membrane stage that uses nanofiltration membranes. Permeate produced by the nanofiltration membrane stage has a high TDS value (total dissolved solids) and is approaching feed water by its ionic composition. Therefore, nanofiltration membrane permeate is forwarded to the inlet to the reverse osmosis facility and mixed with the feed water. This technology enables us to reduce concentrate discharge but does not ensure the reduction in reagent consumption and pretreatment costs. The use of membrane modules with the “open channel” [3] in a number of cases enables us to avoid expensive pretreatment and antiscalant addition. The flow diagram of the membrane facility that uses the “open channel” spiral wound membrane elements [2,3] tailored with low-rejection nanofiltration membranes is presented in Figure 1c. As can be seen in Figure 1c, the membrane facility uses two stages of membrane treatment by nanofiltration membranes instead of one stage of reverse osmosis membranes. To reduce hardness by 20 or 50 times, two stages of nanofiltration membranes are used; each stage reduces the hardness concentration by 5–8 times. Due to this described approach, each membrane stage does not “suffer” from calcium carbonate scaling. The colloidal and organic foulants deposited on the membrane surface are removed using hydraulic flushes. Depending on the conditions, such as TDS, hardness, permeate requirements, etc., the flow diagram of the membrane facility can be changed to fit these conditions. For example, for the case where the hardness value in the permeate should not exceed 50–100 microgram equivalents per liter, high-rejection membranes can be used in the first stage (Figure 1c). This solution again requires the addition of antiscalants into the feed water that enters the second stage. To reduce operational costs and increase reliability, the membrane facility can use not two but three membrane stages furnished with low-rejection nanofiltration membranes (Figure 1d). This technology is used by the authors to provide a reagent-free treatment of stormwater and drainage wastewater for its reuse for boiler feed purposes. The required hardness value lies between 60 and 150 microgram equivalents per liter.

The developed membrane facilities are tailored with spiral wound membrane modules with “open channels” in which the live flow section is increased and hydraulic flow resistance is reduced. This allow us to efficiently remove colloidal and organic foulants by using hydraulic flushing. The “nanoNF” membranes are distinguished by the reduced rejection of divalent ions as well as a substantial difference between divalent and monovalent ion rejection that provides a very low concentration of ions in the “dead areas” in membrane channels and results in reduced scaling compared with other membrane types. The application of low-rejection nanofiltration membranes provides high recovery values of up to 95–97 percent of the feed water flow.

An attempt to evaluate calcium carbonate nucleation and scale growth conditions in the “dead areas” of spiral wound membrane channels was undertaken in [1]. The results of the work showed that calcium carbonate crystal nucleation occurs under high supersaturation levels. In the absence of sufficient supersaturation conditions in membrane channels that usually do not occur when low-rejection nanofiltration membranes are used, it becomes possible to achieve higher recoveries. However, concentrate flow can be reduced by a certain reasonable limit that corresponds to the beginning of the spontaneous nucleation process. When designing a membrane facility, it is crucial to determine the maximum recovery value that prevents calcium carbonate nucleation and crystallization. A method for evaluating this maximum recovery value was described in [1]. However, the determination of this value based on the K parameter corresponding to the onset of scaling yields only approximate results. As already reported [1], the nucleation rate value depends on the supersaturation value. The greater the supersaturation of the solution is, the higher the nucleation rate is and the smaller the formed crystals are. We can evaluate the supersaturation ratio by the sizes of nuclei crystals formed in the flow [1] and detect the beginning of spontaneous nucleation conditions.

The concentrate of the membrane facility can be discharged in the sewer. The cost of discharge depends on the total amount of concentrate on the condition that the total amount of pollution does not exceed the standard amount. Thus, the achievement of minimal possible concentrate flow is very important [18,19,20]. The problem of possibly exceeding the calcium carbonate solubility limits in concentrate and scale deposition in flow measuring instruments and fittings is of great importance.

Experimental research is required to evaluate not only scaling rates but also the minimal concentrate flow rate to avoid crystal nucleation due to supersaturation.

The main goal of the presented research was the economic evaluation of the three possible solutions of the drainage and groundwater treatment for their reuse as boiler feed. The following parameters were determined and evaluated throughout the experiments: conductance, membrane flux, and scaling and organic fouling rates on each membrane stage [18,19,20]. Based on the experimental results, the required operational costs for three proposed and tested flow diagrams were determined, which included antiscalant and cleaning costs, power costs, and membrane replacement costs.

Another important goal of this work was to determine what minimum concentrate value could be achieved for a given feed water composition. The program consisted of three series of experiments. The first series aimed to evaluate membrane rejection and product flow characteristics. The second series was devoted to calculating scaling and organic fouling rates at each membrane stage to evaluate pretreatment and cleaning costs. And in the third series, an attempt was made to determine the minimum concentrate flow rate or maximum recovery value at which calcium carbonate crystallization does not begin.

## 2. Materials and Methods

The first series of experiments was devoted to the investigation of product water quality as a function of recovery on each stage.

Dependencies of TDS, calcium, and bicarbonate concentration values on initial water volume reduction coefficient K values were developed. The comparison of rejection characteristics of different membranes enabled us to select membrane types for the first and second stages to reach the required product quality with lower operational costs.

In the second experimental series, calcium carbonate scaling rates were determined in membrane elements tailored with different types of membranes used at the first and second stages of the membrane facility. Experiments were performed both for the drainage wastewater (Table 1) used as a feed water for stage 2 and for the treatment of the second stage feed water, which was produced as a first stage permeate.

In the third series, the condition of the initiation of spontaneous calcium carbonate nucleation in the concentrate flow was investigated. The maximum recovery value for the first stage membrane module was determined to reach the minimal concentrate flow rate and avoid crystallization.

The experimental procedure is described in a number of publications [1,5].

The conducted experiments aimed to detect the initial volume reduction coefficient K values that correspond to the beginning of scaling in the modules furnished with nanofiltration membranes.

In the first stage nanofiltration membranes, “nanoNF” is used both to produce first stage permeate and to reduce concentrate flow, as shown in Figure 1d. Permeate produced by membrane modules used for recovery increase is forwarded to the inlet of the first stage pump (Figure 1c,d).

To study membrane performance on each stage, we had to control the chemical composition of permeates and concentrates to evaluate supersaturation values and calcium carbonate deposition rates.

**Figure 2:**
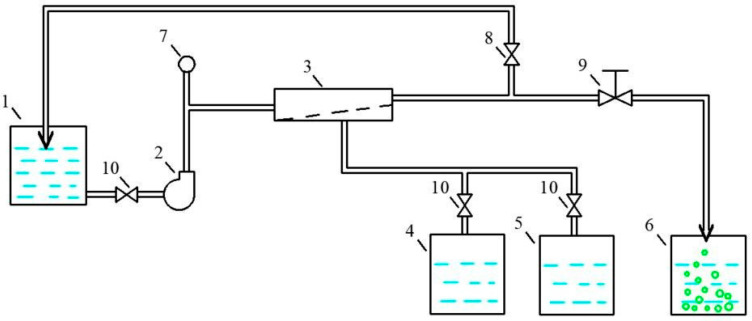
Flow diagram of membrane test unit: 1—feed water tank; 2—rotary pump; 3—membrane spiral wound module in the plastic pressure vessel; 4 and 5—product water collection tanks; 6—tank to collect water after flushing; 7—pressure gauge; 8—pressure regulation valve; 9—hydraulic flushing valve; 10—shut-off valves.

The flow diagram of the membrane test unit is presented in Figure 2. Feed water was added to the feed water tank (1) and injected by the pump (2) to the membrane module (3) in the pressure vessel. In the membrane module, the feed water is separated into two streams: permeate, water, permeated through membrane, and concentrate stream, that contains all the rejected impurities (Figure 2).

Concentrate is returned back to tank 1, and the permeate of the membrane module is forwarded in tanks 4 or 5 depending on the K value. The working pressure is controlled by pressure gauge 7. The permeate produced at K values below 10 is forwarded to tank 4.

The initial volume reduction coefficient K is defined as the ratio of the flow rate of the feed water to the flow rate of the concentrate in the membrane facility. This shows how many times the feed water volume in tank 1 decreased during permeate production.

The permeate collected during the test run under K values in the range between 10 and 40 was collected in tank 5. The first stage permeate collected in tank 4 was used as a feed water for the second stage experiments. To compare the operational parameters of the different membranes used at the first and second stages, experiments were conducted to determine calcium carbonate scaling rates and organic fouling rates. Membrane elements with low-pressure reverse membranes “BLN” and nanofiltration membranes “70NE” supplied by “TORAY Co.” (Japan, Tokyo) were used.

The surface area of the membranes in the spiral wound element type 1812 was 0.5 m^2^.

For comparison, newly developed “nanoNF” membranes (Membranium, Rosnanotech, Vladimir, Russia) were tested. The membrane manufacturers provide the following basic characteristics for nanofiltration membranes: rejection of sodium chloride and rejection of magnesium sulfate. The “70NE” membrane model provides 70% rejection of sodium chloride under a test pressure of 4.1 bar. The basic parameters of the “nanoNF” model provided by “Membranium” demonstrate 60% sodium chloride rejection under the pressure of 7 bar. The experience of using such membranes shows that under the same conditions, “nanoNF” exhibits lower rejection performance.

The method of scaling rates’ determination in membrane modules has been described in a number of publications [1,2,3,4,5]. This method is based on the mass balance of calcium. Calcium concentrations (expressed in millie equivalents/L) in tanks 1, 4, and 5 were determined at various stages of the experiment, along with the K values. The difference between the total calcium amount in feed water tank 1 at the beginning of the experimental test run and amount of calcium carbonate (millie equivalents) in tanks 4 and 5, calculated as the product of calcium concentration on the solution volume, at the certain K value, corresponds to the amount of calcium carbonate deposited in the membrane element. To calculate the scaling rate value, the amount of deposited calcium should be calculated, and the dependences of the calcium carbonate amount deposited on the membrane on K and time should be built. The value of the time derivative of the dependence of calcium carbonate mass on time at different points in the experiment corresponds to the value of calcium carbonate formation. Graphically, this can be determined as the tangent to the graph of the dependence of calcium amount on time.

The third series of the experiments was devoted to the investigation of calcium carbonate nucleation in the concentrate flow. In this series, samples of the material deposited on the membrane surface or formed in the concentrate flow were prepared for their further investigation using SEM techniques [1]. Concentrate that contained organic foulant particles and formed nuclei crystals was filtered through microfiltration membrane MFAS-OS-3 and then placed in the drying cabinet, where they were kept at a temperature of 50 °C. The deposits were investigated using SEM (scanning electronic microscopic) techniques. Scanning electron microscope “Quanta 250” (produced by FEI Company (Hillsboro, OR, USA) with a thermo emission cathode was used. The microscope was supplied with the system of energy-dispersive X-ray analysis “GENESIS APEX 2 EDS System with Apollo X SDD EDAX”. The chemical composition of the deposits and observations were investigated in “low vacuum” mode conditions at accelerating voltages of 12.5 and 15 kilovolts.

## 3. Discussion of the Results

### 3.1. Results of the First Series of Experiments

**Figure 3:**
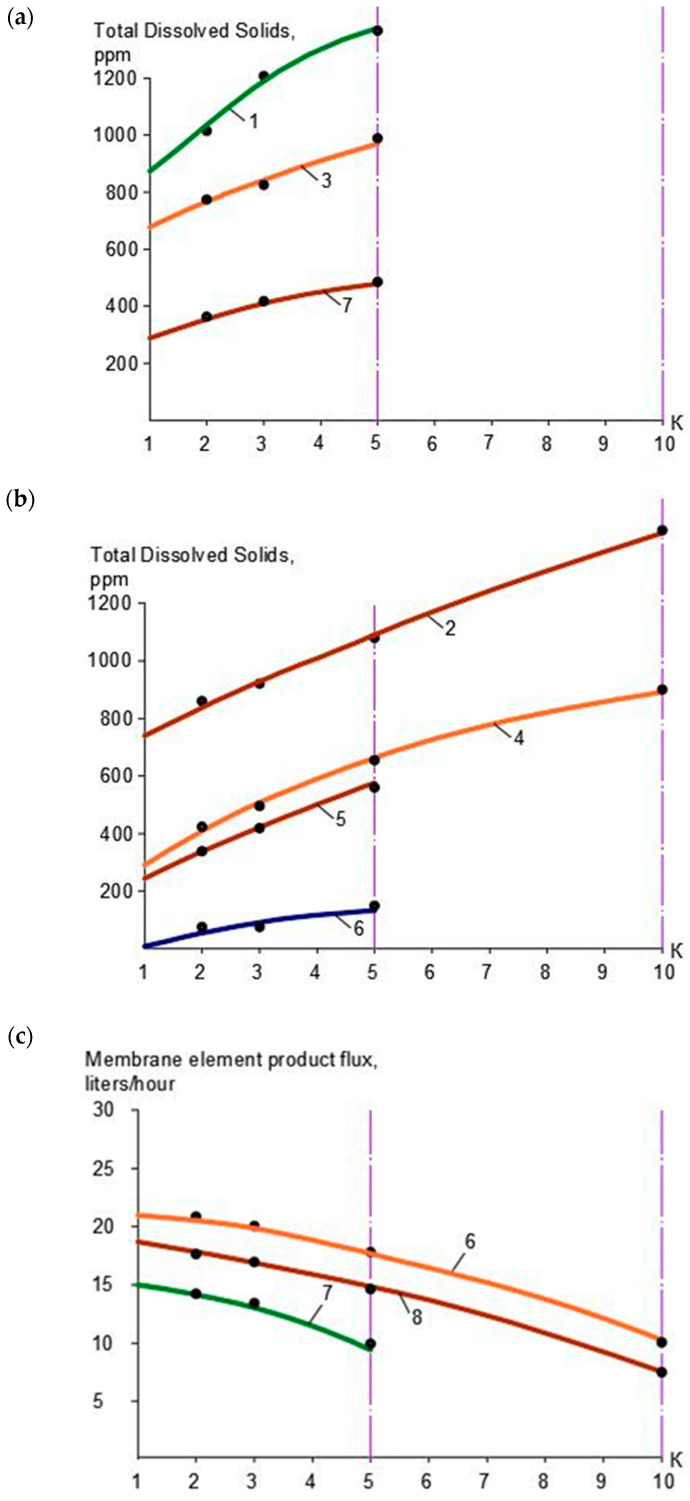
Results of experiments with “ nanoNF“ membranes in the first stage as well as “BLN” and “nanoNF” membranes in the second stage: (**a**) TDS concentration versus K, the first stage; (**b**) TDS concentration versus K; (**c**) membrane element product flow vs. K; 1—concentrate, first stage, “nanoNF” membranes; 2—concentrate, second stage, “nanoNF” membranes; 3—permeate, first stage,”nanoNF” membranes; 4—permeate, second stage, “nanoNF” membranes; 5—concentrate, second stage, “BLN” membranes; 6—permeate, second stage, “BLN” membranes; 7—“nanoNF” membranes, first stage; 8—“BLN” membranes, second stage.

**Figure 4:**
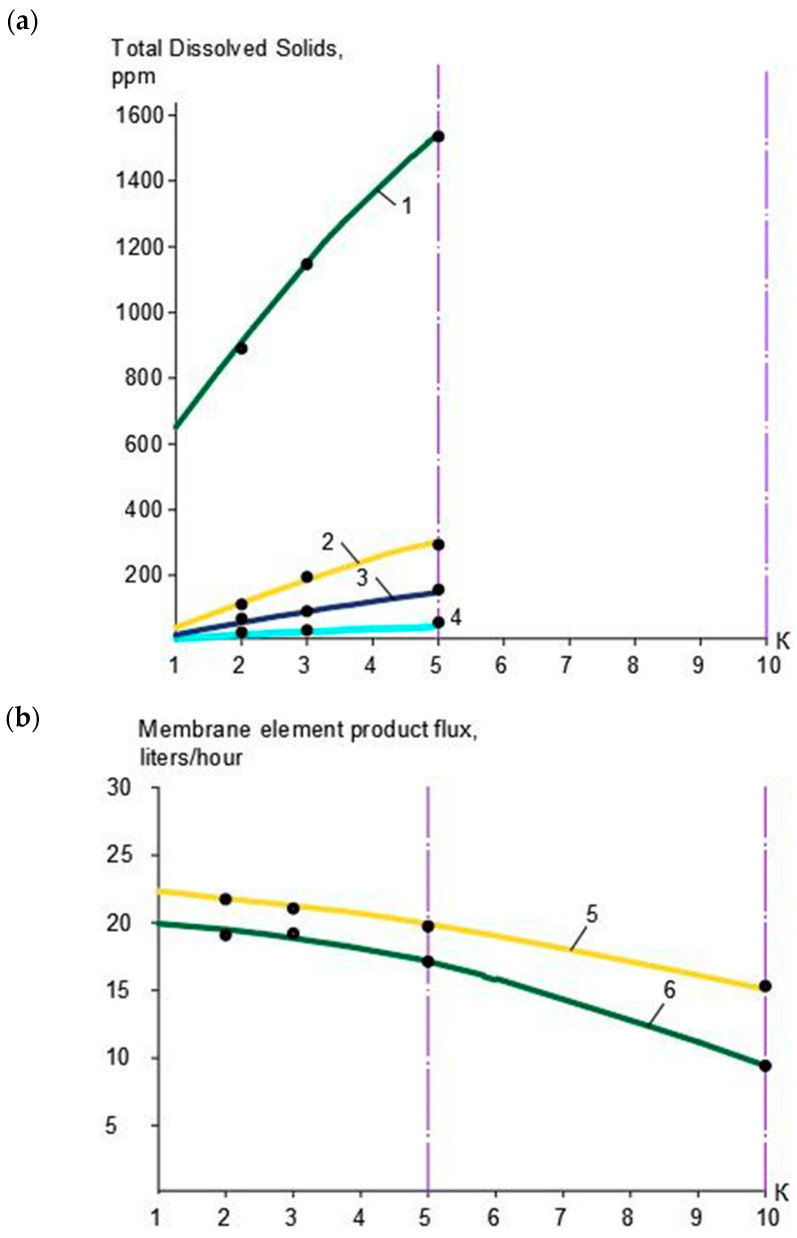
Results of experiments with “70NE” membranes in the first and in the second stage: (**a**) TDS concentration versus K; (**b**) membrane element product flow vs. K; 1—concentrate, first stage; 2—concentrate, second stage; 3—permeate, first stage; 4—permeate, second stage; 5—product flow, first stage; 6—product flow, second stage.

In the first series, the use of nanofiltration membranes of models “nanoNF” and “70NE” was tested and compared. In the first stage, TDS and color values, as well as calcium, magnesium, sulfate, chloride, and ammonia ion concentration values, in concentrate and permeate depending on the initial volume reduction coefficient K values, were determined, as shown in Table 1. Dependencies of TDS values in the concentrate and permeate of “nanoNF” membranes in the first and the second membrane stages are shown in Figure 3a, and similar dependencies obtained with “70NE” membranes are presented in Figure 4a. The reduction in the “nanoNF” and “BLN” membrane product flows with coefficient K value growth in the first and second stages of the water treatment is presented in Figure 3b, and the reduction in the “70NE” membrane product flow as a function of the coefficient K value is shown in Figure 4b.

As seen in Figure 3 and Figure 4, the use of nanoNF membranes allows to reduce the feed water TDS by 2–3 times and reduce hardness by 8–10 times and reduce color by more than 10 times. The use of “nanoNF” membranes demonstrated the obtained total hardness value of 0.45 milliequivalents per liter achieved in the second stage. To reach the required value of 0.15 milliequivalents per liter, the use of the third membrane stage is required. It is obvious that to reach the required hardness value, a low-pressure reverse osmosis membrane (“BLN” type) can be efficiently used in the second stage (Figure 3a). The use of “BLN” membranes provides a hardness value of less than 0.1 milliequivalent per liter. It is also well known [1,2,5,10,20] that the use of high-rejection reverse osmosis membranes, despite high efficiency, has a serious disadvantage: the scaling hazard. This requires measures to predict and control scale formation and growth that also increase the operational costs. To control scale formation, various antiscalants are used [1,4,5,14]. Calcium carbonate scaling rates were determined throughout experiments conducted in the first and second series using the test unit shown in Figure 3. The experimental procedure and methods of the results’ processing were developed by other authors and presented in previous publications [1].

The dependencies of the TDS values in the permeate and concentrate of “nanoNF”, “70NE”, and “BLN” membranes used in the first membrane stage on the value of coefficient K are presented in Figure 3a. Figure 3b demonstrates the reduction in the “BLN” and “nanoNF” membrane permeabilities in the first stage with the growth of K values. Figure 4 shows the rejection characteristics (a) and permeabilities (b) of “70NE” membranes used in the first and second stages as a function of the K value.

**Figure 5:**
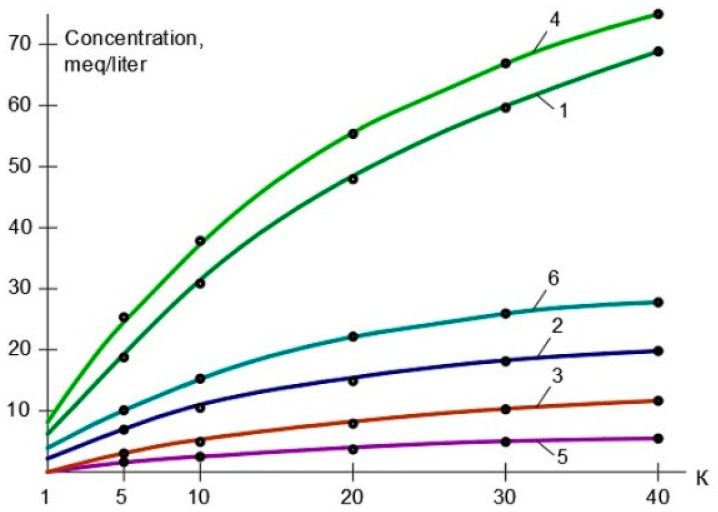
Dependencies of calcium concentration on K during feed water concentration with different membranes: 1—concentration of calcium in concentrate of “nanoNF” membranes; 2—concentration of bicarbonate ions in concentrate of “nanoNF” membranes; 3—concentration of calcium in permeate of “nanoNF” membranes; 4—concentration of calcium in concentrate of “70NE” membranes; 5—concentration of calcium in permeate of “70NE” membranes; 6—concentration of bicarbonate ions in concentrate of “70NE” membranes.

Figure 4 shows the experimentally obtained dependencies of calcium concentrations on K values in the first stage (Figure 4a) and in the second stage (Figure 4b). For comparison, the experimental results were obtained using nanofiltration membranes “nanoNF” and “70NE”. As shown in Figure 5, the nanofiltration “70NE” membrane possess higher rejection, which results in lower TDS and calcium concentration values in permeate, scaling hazard, and the necessity to add antiscalants to the feed water.

In the first series, the use of nanofiltration 70NE membranes was also evaluated on both the first and second stages of the membrane facility (the scheme shown in Figure 1c). The reason for this was the assumption that “70NE” membranes have higher rejection characteristics than “nanoNF” membranes and demonstrate higher performance. The results of the experiments are shown in Figure 3, Figure 4 and Figure 5. As can be seen in the plots shown in Figure 5, the second stage permeate by hardness corresponds to the requirements for the boiler feed water, which lies between 150 and 600 microgram equivalents per liter. But we should also take into account that the treatment of natural water with high hardness values using “70NE” membranes raises the concern of calcium carbonate scaling in membrane modules. Despite the fact that “70NE” membranes have lower rejection characteristics than “BLN” reverse osmosis membranes, scale formation in “70NE” membrane modules still takes place, and scaling rates are 2–3 times lower than in “BLN” membrane modules [1,12,13]. Nevertheless, the operation of membrane facilities furnished with nanofiltration membranes “70NE” still requires antiscalant dosing and the application of timely cleanings to remove the scale that causes increases in operation costs.

### 3.2. Results of the Second Series of Experiments

**Figure 6:**
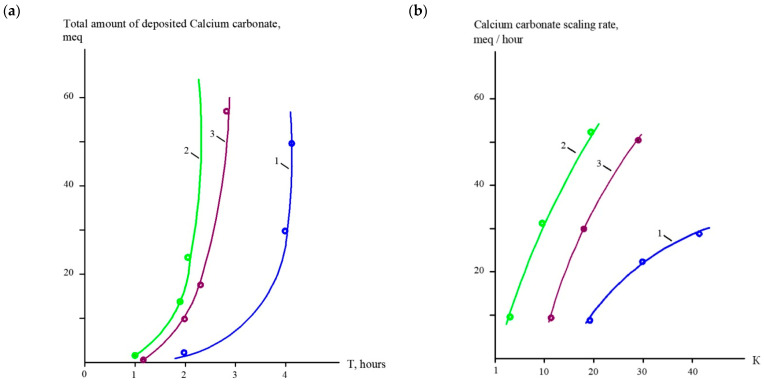
Evaluation of calcium carbonate scaling rates: (**a**) dependencies of accumulated calcium on time of experiment; (**b**) dependencies of scaling rate values on K. 1—experiment with “nanoNF” membranes; 2—experiment with “70NE” membranes; 3—“70NE” membranes with antiscalant addition.

Figure 6 demonstrates the main steps used to evaluate scaling rates in the spiral modules: the dependencies of the accumulated calcium amount on the time of the experiment (Figure 6a) and determining the scaling rate values on K values (Figure 6b). The value of the scaling rate is determined as a tangent of the function, shown in Figure 6a.

**Figure 7:**
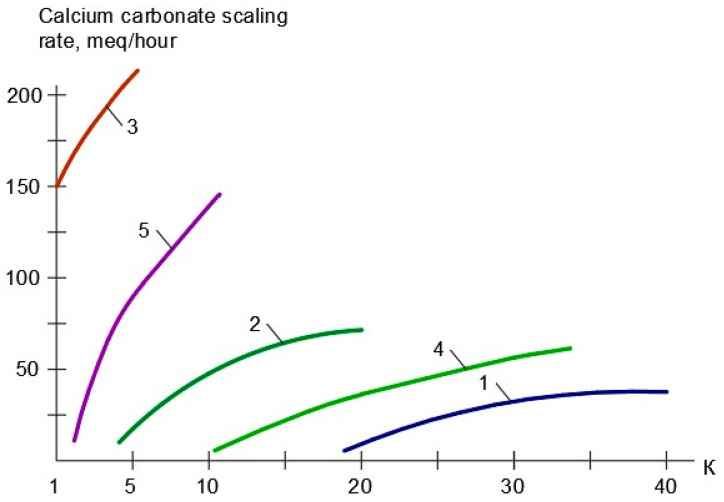
Calcium carbonate scaling rates versus K relationships obtained for different membrane types: 1—“nanoNF” membranes; 2—“70NE” membranes; 3—low-pressure reverse osmosis membranes “BLN”; 4—“70NE” membranes with “Aminat-K” antiscalant dosing in the feed water; 5—“BLN” membranes with “Aminat-K” antiscalant dosing in the feed water.

Figure 7 demonstrates the results of the evaluation of the calcium carbonate formation rates in membrane modules as a function of the initial volume reduction coefficient K. A comparison of the calcium carbonate scaling rates in membrane modules with different membranes is presented for the case that feed water is treated in the first stage. Feed water composition is presented in Table 1.

**Figure 8:**
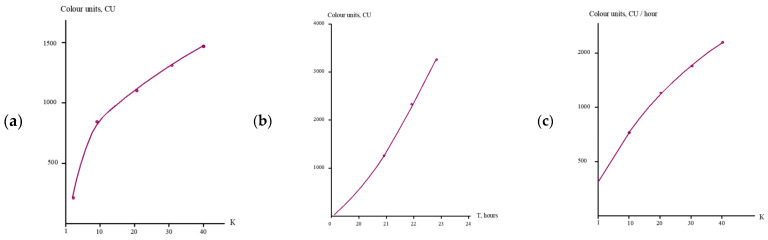
Main steps to evaluate organic fouling by NOM: (**a**) color versus K relationship; (**b**) mass of adsorbed organics versus time; (**c**) organic fouling rate versus K values.

As can be seen in Figure 7, the use of “70NE” membranes results in the increase in the scaling rate in membrane modules as compared to the use of “nanoNF” membranes. Organic fouling rates were determined similarly, as shown in Figure 8.

### 3.3. Results of the Third Series of Experiments

**Figure 9:**
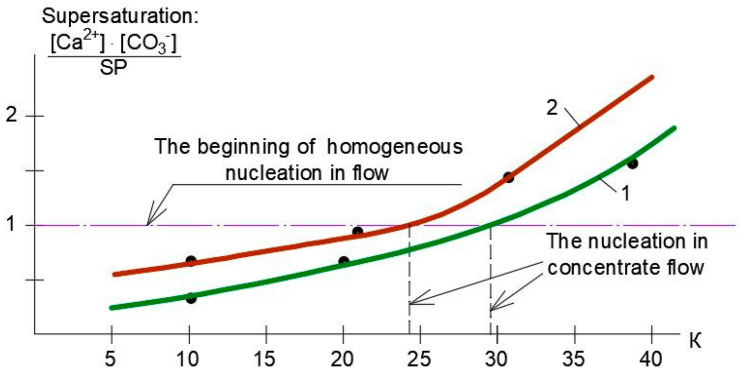
Dependencies of supersaturation ratio values on K values and determination of calcium carbonate nucleation start threshold: 1—experiments with “nanoNF” membranes; 2—experiments with “70NE” membranes.

To investigate the conditions of calcium carbonate nucleation in concentrate flow and evaluate supersaturation values, the relationships of supersaturation and K values were developed, as shown in Figure 9. The main steps to determine calcium carbonate supersaturation values are presented in Table 2. The supersaturation value is determined as the ratio of the product of calcium and carbonate ion concentrations to the solubility product (SP) value. The solubility product is an equilibrium constant that represents a product of ion concentrations at the crystallization threshold.

Previously conducted research [1,7] revealed that calcium carbonate homogeneous nucleation is observed in concentrate flow at the supersaturation ratio level value of 1.0 × 10^−5^.

As can be seen in Figure 9, treatment of the feed water (Table 1) using “nanoNF” membranes at K values equal to 29–30 supersaturation ratio values exceeds 1.0 × 10^−5^, which corresponds to the beginning of nucleation in concentrate flow.

In the case when “70NE” membranes are applied, the homogeneous crystallization begins after a K value of 20 is reached.

The beginning of nucleation is illustrated by the presence of crystals in concentrate flow.

During the experiment using “70NE” membranes, concentrate samples were taken at K values of 20 and 30. Concentrate samples were filtered using microfilter and studied using a scanning electronic microscope.

**Figure 10:**
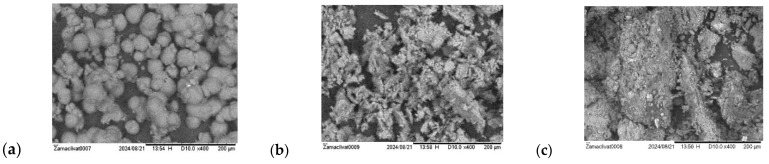
SEM photos of deposits extracted from the concentrate flow during experiments with “nanoNF” membranes: (**a**) K = 30; (**b**) K = 40; (**c**) after hydraulic flushing performed at K = 40.

SEM photos of the filtered deposits taken at K = 20 and K = 30 are presented in Figure 10a,b.

As can be seen in the photo, the withdrawn foulant does not show any presence of calcium carbonate crystals. But the results of the chemical element composition analysis performed using SEM revealed that the deposit contains more than 50 per cent of calcium carbonate.

The feed water contains natural organic material (NOM), which is confirmed by high color values (Table 1). Therefore, organic material does not allow us to see crystals. Large amounts of organic material in the deposit is also confirmed by the high carbon concentration value in the sediment. By the end of each conducted test run after a K value of 40 was achieved, hydraulic flushing was performed. The hydraulic flushing procedure was initiated by the opening of valve 7. Hydraulic hammer occurred due to rapid pressure reduction, where the transit cross flow through the membrane element increased and the concentrate flow swept all of the foulants collected on the membrane surface. After flushing, the concentrate sample was collected, filtered, and analyzed using SEM techniques. The photo of the sediment removed from the membrane surface is presented in Figure 10c.

The analysis results show that calcium carbonate holds no more than 10 per cent of the total sediment weight. The main weight of the deposit is formed by natural organic materials (NOMs), which are humic and fulvic acids. To solve the technological problem of reaching 50 microgram equivalent/L of hardness in the product water, three types of flow diagrams were investigated:

1. “NanoNF” membranes in the first stage and “BLN” membranes in the second stage;
2. “70NE” membranes in the first stage and in the second stage;
3. Three stages of the “nanoNF” membranes used to reach the required water quality for boiler feed without antiscalant use.

### 3.4. Processing of the Results: Feasibility Study of the Developed Technology

**Figure 11:**
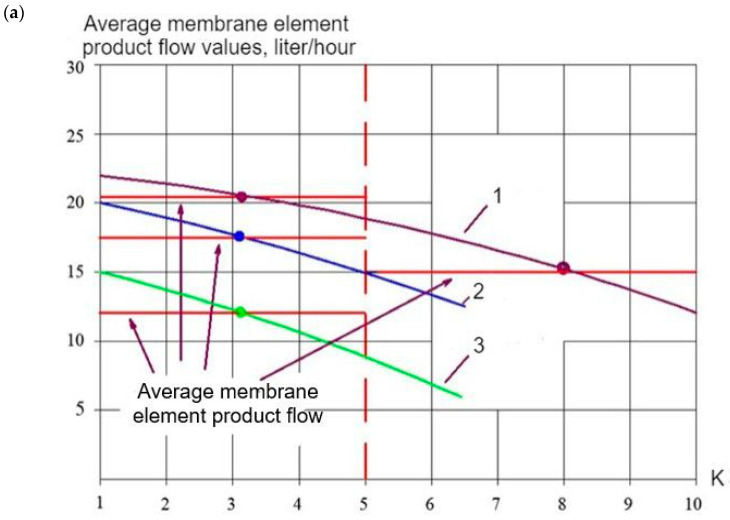

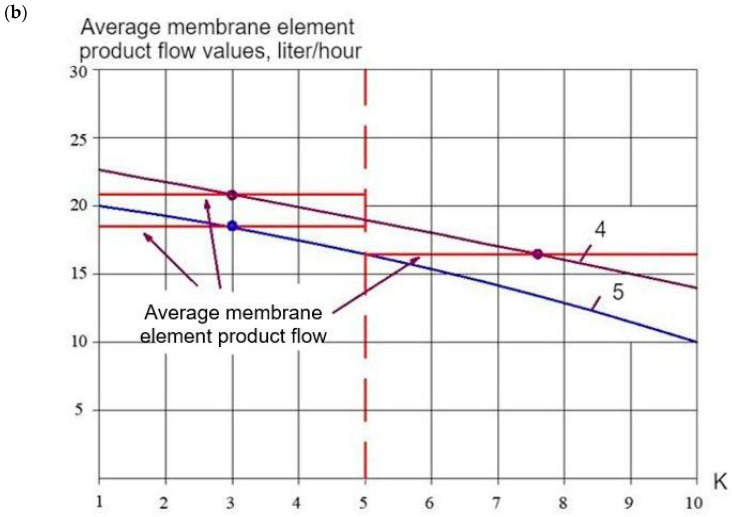
Evaluation of membrane surface and number of membrane elements required to produce designed product flow rate and reach required recovery: (**a**) for the first stage; (**b**) for the second stage; 1—“nanoNF”, first stage; 2—“BLN”, first stage; 3—“BLN”, second stage; 4—“70 NE”, first stage; 5—“70NE”, second stage.

For membrane facility project development and operational costs evaluation, we selected the membrane types used at each membrane stage and calculated the membrane surface area and the number of membrane elements used on each membrane stage. Figure 11a demonstrates the principles of the calculation of the required area on “nanoNF”, “70NE”, and “BLN” membranes based on the results of the first series of experiments shown in Figure 3b and Figure 4b. Using two ranges of K change (from 1 to 5 and from 5 to 10), we select the average product flow value. For the selected flow diagram shown in Figure 1c, we made a calculation for the facility of 10 m^3^/h capacity. To make the calculation, all the flow values shown in Figure 1c were divided by 10. Assuming that the average membrane surface in 1812 standard membrane elements is 0.5 m^2^, we divide the required product flow value by the product flow value and obtain the required “nanoNF” membrane surface in the first stage. Similarly, the surface of the “nanoNF” and “BLN” membranes in the second stage was calculated.

Table 3 shows the results of the calculation of the 8040 membrane element numbers in each stage. The selection of the membranes was made based on the experimental results (Figure 3 and Figure 4).

Three schemes were evaluated, and operation costs were compared:

1. “nanoNF” membranes are used in the first stage and “BLN” membranes are used in the second stage. This can be recognized as the best solution to produce water for boiler feed with hardness not exceeding 150 microgram equivalent/L. But the necessity to add antiscalant and apply timely cleanings to remove calcium carbonate increases operational costs and reduces the reliability of the scheme as well.
2. The scheme with nanofiltration membranes “70NE” was used both in the first and in the second stages. This technology also requires antiscalant addition in the first stage due to the high hardness of the feed water. But the scaling rates in “70NE” membrane modules are substantially lower than those in modules tailored with “BLN” membranes. Nanofiltration membranes are operated under the pressure of 8 bar, which provides a substantial reduction in power consumption.
3. To ensure “reagent-free” technology without antiscalant addition, a three-stage scheme is designed (Figure 1d). The use of “nanoNF” membranes in each stage is applied, excluding the formation of a calcium carbonate scale in membrane modules in a wide range of feed water compositions.

To compare the operational costs of the schemes, we have to determine the following [1,14,19,20]:

- Annual power consumption costs;
- Annual membrane replacement costs for replacement every 5 years;
- Annual antiscalant costs;
- Annual cleaning chemical costs.

Table 3 contains calculations of the main operational costs for the membrane facility with 10 m^3^/h capacity using one of the three compared technological schemes discussed above.

Power costs are calculated based on the power consumption of the working pumps to provide the required water flow and pressure. To provide 16 bar specific power consumption, 1 kilowatt–hour is required. To provide 12 bar, the specific power consumption is 0.75 kilowatt–hour, and for 7 bar, the specific power consumption is 0.5 kilowatt–hour. The cost of 1 kilowatt–hour is assumed to be USD 0.05. For power cost calculations, the electricity tariffs accepted in Russia were used. Prices for membranes and reagents are taken from the websites of the companies selling them. The average cost of the membrane element of 8040 standard is taken to be USD 500. The membrane element of 8040 standard contains, on average, 40 m^2^ of membranes. The average antiscalant dose is 0.01 g/L of feed water. For the case when “70NE” membranes are used in the first stage, the antiscalant dose is reduced to 0.003–0.005 g/L of the feed water. Water flow values that enter the first and second stages are determined using Figure 1.

## 4. Conclusions

A new reagent-free technology is developed to treat and reuse drainage wastewater instead of discharging them into surface water sources.

The proposed technology allows for wastewater to be treated without the use of pretreatment reagents and antiscalants with reduced concentrate discharge.

Concentrate flow reduction is very important when the amount of sewer discharges is accounted for. Therefore, determination of the maximum recovery value that does not initiate nucleation in the flow is an important step in the industrial application of membrane systems. Once the supersaturation ratio is exceeded, nucleation occurs in the concentrate flow, leading to crystal deposition on fitting surfaces and on the rotameter floats and water meters.

## Figures and Tables

**Table 1:** Chemical composition of the drainage wastewater.

N	Component	Drainage Wastewater
1	Calcium, ppm	6.7
2	Magnesium, ppm	0.8
3	Total hardness	7.5
4	Chlorides, ppm	55
5	Sulfates, ppm	180
6	Bicarbonates, ppm	2.6
7	Sodium + potassium, ppm	46
8	pH	7.5
9	Ammonium, ppm	6.5
10	Oil product, mg/L	0.2
11	Color units, CUs	149
12	TDS, ppm	810

**Table 2:** Results of evaluation of supersaturation ratio values and homogeneous nucleation start threshold in the concentrate flow.

N	K	Ca^2+,^ meq/L	HCO_3_^−^, meq/L	pH	TDS, ppm	Solubility Product, SP, (meq/L)^2^	[CO_3_^2−^]/[HCO_3_^−^]	[CO_3_^2−^], meq/L	$\frac{\left[{Ca}^{2+}\right]\;\left[{CO}_{3}^{2-}\right]}{\mathrm{SP}}$ (Supersaturation)
“nanoNF” membranes									
1	10	31	11	7.7	2400	5.22 × 10^−9^	0.3	3.3	0.2 × 10^5^
2	20	42	15	8	3300		0.4	7	0.6 × 10^5^
3	30	61	20	8.1	4400		0.4	8.1	1.1 × 10^5^
4	40	70	22	8.2	5200		0.5	10	1.4 × 10^5^
“70NE” membranes									
5	10	38	15	7.7	3000		0.3	5	0.4 × 10^5^
6	20	55	20	8	4000		0.4	8.4	0.8 × 10^5^
7	30	65	24	8.1	5100		0.5	10	1.3 × 10^5^

**Table 3:** The results of capital and annual operational costs evaluation for the three applications of membranes in water treatment schemes. Calculations were performed for the case of drainage water treatment (Table 1) with 10 m^3^/h capacity.

№	Components of Operating Costs	Flow Diagram 1	Flow Diagram 2	Flow Diagram 3
		I stage—“nanoNF” II stage—“BLN”	I stage—“70NE” II stage—“70NE”	I stage—“nanoNF” II stage—“nanoNF” III stage—“nanoNF”
**1.**	**Electric power costs**	(Feed water flow, (m^3^/h) × specific power consumption, (kWh/m^3^) × 7000 h/year × 0.05 USD/kW)		
1.1.	I stage	14.5	14.5	15.5
1.2.	II stage	12.0	12.0	13.0
1.3.	III stage			12.0
**1.4.**	**Annual electric power costs, USD/year**	**5687.50**	**4637** **.50**	**7087.50**
**2.**	**Membrane replacement costs**	**Number of elements × 500/5**		
2.1.	Number and model of elements in the first stage	14.5:0.04:40 = 9 elements nanoNF 8040	14.5:0.36:4 = 10.06 elements 70NE 8040	15.5:0.4:4 = 9.68 elements nanoNF 8040
2.2.	Number and model of elements in the second stage	12.0:0.24:4 = 12.5 elements BLN 8040	12.0:0.44:4 = 6.8 elements 70NE 8040	13.0:0.44:4 = 7.38 elements nanoNF 8040
2.3.	Number and model of elements in the third stage			12.0:0.5:4 = 6 nanoNF 8040
**2.4.**	**Annual membrane replacement costs, USD/year**	**(9 + 13) × 500/5 = 2200.00**	**(11 + 7) × 500/5 = 1800.00**	**(10 + 8 + 6) × 500/5 = 2400.00**
**3.**	**Antiscalant annual costs evaluation, USD**	**Antiscalant consumption, kg/h × 7 000 h × 12 USD/kg**		
3.1.	Antiscalant dose, kg/h	(Feed water flow, (m^3^/h) × dose, kg/m^3^)		
3.2.	I stage	–	5	–
3.3.	II stage	10	–	–
3.4.	III stage			–
**3.5.**	**Antiscalant annual costs, USD**	**10,080.00**	**6048.00**	**–**
**4.**	**Cleaning costs, USD/year**	**Number of** **elements × 2 kg/element × 10 USD/kg × number of cleanings per year**		
4.1.	Cleaning chemical consumption: 2 kg/element			
4.2.	I stage	9 × 2 × 10 × 3 = 540	11 × 2 × 10 × 4 = 880	10 × 2 × 10 × 3 = 600
4.3.	II stage	13 × 2 × 10 × 6 = 1 440	7 × 2 × 10 × 2 = 280	–
**4.4.**	**Annual cleaning costs, USD**	**1980.00**	**1160.00**	**600.00**
**5.**	**Total annual costs, USD/year**	**19,949.50**	**13,645.50**	**7927.50**

## Data Availability

The data are available in publications.
